# Graph theoretical analysis of brain connectivity in phantom sound perception

**DOI:** 10.1038/srep19683

**Published:** 2016-02-02

**Authors:** Anusha Mohan, Dirk De Ridder, Sven Vanneste

**Affiliations:** 1 Lab for Clinical & Integrative Neuroscience, School of Behavioral and Brain Sciences, The University of Texas at Dallas, USA; 2 Department of Surgical Sciences, Section of Neurosurgery, Dunedin School of Medicine, University of Otago, Dunedin, New Zealand

## Abstract

Tinnitus is a phantom sound commonly thought of to be produced by the brain related to auditory deafferentation. The current study applies concepts from graph theory to investigate the differences in lagged phase functional connectivity using the average resting state EEG of 311 tinnitus patients and 256 healthy controls. The primary finding of the study was a significant increase in connectivity in beta and gamma oscillations and a significant reduction in connectivity in the lower frequencies for the tinnitus group. There also seems to be parallel processing of long-distance information between delta, theta, alpha1 and gamma frequency bands that is significantly stronger in the tinnitus group. While the network reorganizes into a more regular topology in the low frequency carrier oscillations, development of a more random topology is witnessed in the high frequency oscillations. In summary, tinnitus can be regarded as a maladaptive ‘disconnection’ syndrome, which tries to both stabilize into a regular topology and broadcast the presence of a deafferentation-based bottom-up prediction error as a result of a top-down prediction.

The brain can be considered as a complex adaptive system, analogous to the economy, an ant society, or the internet. Under general conditions, complex dynamics can be generated by systems fulfilling the following two requirements: (1) the presence of noise (e.g. 1/f dynamics) and (2) a small-world topology^1^. Complex adaptive systems are dynamic systems that are able to adapt to and evolve with a changing environment. The changes brought about in the system helps it to either maintain or improve its function in order to sustain and survive in the particular environment (i.e. persist as an organized system). Complex adaptive systems are characterized by emergence^2^,^3^, and it has been proposed that tinnitus is an emergent property of interacting tinnitus-related networks^4^.

Complex adaptive systems are characterized by the presence of noise. The phenomenon of 1/f structure also known as pink noise or inverse-power-law spectra from the brain dynamics is widely recognized^5^,^6^. It was demonstrated that both MEG and EEG recordings of spontaneous neural activity in humans display 1/f-like power spectra, suggesting that the power-law scaling arises from self-organized neural oscillatory networks in the brain^7^. This is a result of internal interactions between parts of the brain and is probably required for optimal processing of information^8^. Studies have shown in several brain disorders, such as schizophrenia^9^, anxiety^10^, autism^11^, Alzheimer^8^ and epilepsy^12^, this 1/f-like power spectra shift towards white (1/f^0^) or Brownian (1/f^2^) noise. This shift to more randomness in the case of 1/f^0^ may imply less coordinated signal organization or more random processing of information.

It is widely agreed upon that the organization of the human brain connectome is characterized by a small-world topology^13^,^14^,^15^,^16^,^17^,^18^,^19^,^20^, with high efficiency of information transfer amongst neighboring and long-distant nodes^17^,^21^. Small worldness is a characteristic given to a type of mathematical graph in which most nodes are not neighbors of one another, but most nodes can be reached from every other node by a small number of connections. Imaging studies applying network science to the analysis of the functional connectivity of brain networks in different pathologies have reported consistent evidence of changes in network properties and deviation from small-worldness^22^. Increased path length due to reduced long-distance connectivity and decreased clustering respectively leading to decreased global and local efficiency of information transfer have been reported in Parkinson’s disease, traumatic brain injury, fronto-temporal dementia and Alzheimer’s disease^22^. Bassett and colleagues (2008) report low clustering and disruption of long-distance connections in schizophrenia patients relative to the controls.

In this study, we investigate whether tinnitus patients and controls show distinct topological patterns. Tinnitus is the perception of a sound in the absence of a corresponding auditory input from the environment and therefore is also considered as a phantom sound^4^,^23^. The most common etiology of tinnitus is deafferentiation accompanied with or without hearing loss. It has been suggested that the unified percept of tinnitus could be considered an emergent property of dynamically changing networks, each representing a specific characteristic of the tinnitus (sidedness, loudness, distress, mood, tonality, etc.) with a specific spontaneous oscillatory pattern and functional connectivity signature^4^. These subnetworks could hypothetically integrate in a rich club, which connects the dominant hubs of each subnetwork^24^,^25^. It is known that tinnitus is characterized by spontaneous changes in auditory and non-auditory brain areas^26^. A recent study of resting-state source localized EEG with tinnitus subjects has pointed to the existence of multiple distributed sub-networks with partially overlapping brain areas^27^. In parallel with other brain-related disorders mentioned above, we hypothesize that tinnitus patients show diminished neural flexibility, i.e. a loss of small worldness, characterized by significant changes in the power scale and network topology. The identification of specific oscillatory patterns and connectivity signatures for tinnitus might further explain the underlying neurophysiological mechanism and, as a result, help in the identification of a treatment, since no treatment exists for this auditory phantom phenomenon to date^28^.

## Materials and Methods

### Patients with an auditory phantom percept

The patient sample consisted of 311 patients (*M* = 50.63 years; *SD* = 13.67; 210 males and 101 females) with continuous tinnitus. If the onset of the tinnitus dated back a year or more, the patient’s condition was considered chronic. In order to increase the homogeneity of the sample, individuals with pulsatile tinnitus, Ménière’s disease, otosclerosis, chronic headache, neurological disorders, such as brain tumors, and individuals being treated for mental disorders were excluded from the study. All patients reported the perceived location of their tinnitus as well as the type of tinnitus. The pure tone audiometric thresholds at 0.125 kHz, 0.25 kHz, 0.5 kHz, 1 kHz, 2 kHz, 3 kHz, 4 kHz, 6 kHz and 8 kHz were obtained using the British Society of Audiology procedures^29^. In addition, the pitch and loudness of the perceived tinnitus were measured by performing a simple tinnitus analysis contralateral to the tinnitus ear in patients with unilateral tinnitus and contralateral to the worst tinnitus ear in patients with bilateral tinnitus. A 1 kHz pure tone was presented contralateral to the (worst) tinnitus ear at 10 dB above the patient’s hearing threshold in that ear. The frequency of the tone was adjusted until the pitch of the tone matched the perceived pitch of the patient’s tinnitus. The intensity of this tone was then adjusted in a similar way until it corresponded to the perceived loudness of the patient’s tinnitus. The tinnitus loudness (dB SL) was computed by subtracting the absolute tinnitus loudness (dB HL) from the audiometric threshold at that frequency^30^,^31^. See Table 1 for an overview of the tinnitus characteristics.

This study was approved by the local ethical committee (Antwerp University Hospital) and was in accordance with the declaration of Helsinki.

### Healthy control group

A healthy control group (N = 256; *M* = 49.514 years; *SD* = 14.82; 154 males and 102 females) was included in the study. None of these subjects were known to suffer from tinnitus. Subjects with psychiatric or neurological illness, history of psychiatric or drug/alcohol abuse, history of head injury (with loss of consciousness) or seizures, headache, or physical disability were excluded from the study. No hearing assessment was performed for these healthy controls.

### Data collection

Collection of the data was under approval of IRB UZA OGA85. All patients gave an informed consent. Continuous resting state EEG data was obtained from both subject groups in an eyes closed condition for five minutes (sampling rate = 500 Hz, band passed 0.15–200 Hz). Recordings were obtained in a fully lighted room with each participant sitting upright on a small, but comfortable, chair. The EEG was sampled using Mitsar-201 amplifiers (NovaTech http://www.novatecheeg.com/) with 19 electrodes placed according to the standard 10-20 International placement (Fp1, Fp2, F7, F3, Fz, F4, F8, T7, C3, Cz, C4, T8, P7, P3, Pz, P4, P8, O1, O2) referenced to digitally linked ears. Impedances were maintained below 5 kΩ. Off-line analysis of the data included re-sampling at 128 Hz and band-pass filtering in the range 2–44 Hz. The data was subsequently transposed into Eureka! software^32^, where it was carefully plotted and manually inspected for artifacts. All episodic artifacts including eye blinks, eye movements, teeth clenching, body movement, or ECG artifact were removed from the EEG. Average Fourier cross-spectral matrices were computed for frequency bands delta (2–3.5 Hz), theta (4–7.5 Hz), alpha1 (8–10 Hz), alpha2 (10–12 Hz), beta1 (13–18 Hz), beta2 (18.5–21 Hz), beta3 (21.5–30 Hz) and gamma (30.5–44 Hz). These frequency bands are based on previous research in tinnitus^33^,^34^,^35^,^36^,^37^,^38^. In addition, we calculated the average Fourier cross-spectral matrices for all frequency separately from 2 to 44 Hz.

### Source localization

Standardized low-resolution brain electromagnetic tomography (sLORETA; Pascual-Marqui, 2002) was used to estimate the intracerebral electrical sources. As a standard procedure, a common average reference transformation^39^ was performed before applying the sLORETA algorithm. sLORETA computes neuronal activity in current density (A/m^2^) without assuming a predefined number of active sources. The solution space used in this study and associated lead field matrix are those implemented in the LORETA-Key software (freely available at http://www.uzh.ch/keyinst/loreta.htm). This software implements revisited realistic electrode coordinates and the lead field by applying the boundary element method on the MNI-152 (Montreal neurological institute, Canada) template. The sLORETA-key anatomical template divides and labels the neocortical (including hippocampus and anterior cingulate cortex) MNI-152 volume in 6,239 voxels each of size 5 mm^3^, based on probabilities returned by the Daemon Atlas.

The analyses procedures, henceforth, were performed for both the groups on the average EEG data at sensor level (19 electrodes) and on average EEG data that was source-localized to a specific set of regions of interest (84 Brodmann areas).

### 1/f dynamics

**Figure 1:**
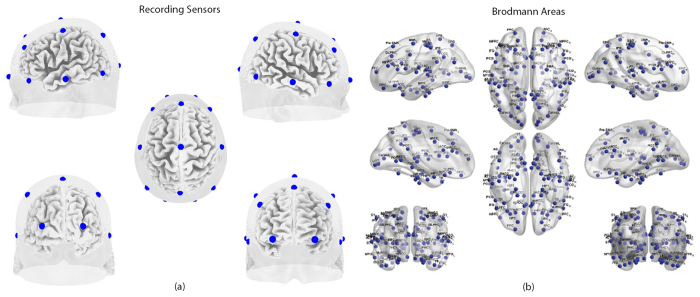
(**a**) 19 recording electrodes used in the study. (**b**) Brodmann areas included in this study.

The power spectrum (PS) of biological time series (an electroencephalogram recording, for instance) often shows a relationship of decreasing power as a function of frequency (f) according to the general equation: PS(f) = ψ * f^*-α*^^40^.The exponent α, therefore, represents the rate at which the power spectrum decreases as a function of frequency, and gives an estimate about the length (or “distance”) of the linear correlations within the time series in question. In other words, the slope of the power spectrum provides an index of “temporal memory effects” in the time series^41^. White noise, for instance, has no correlation over time (its autocorrelation is represented by a Dirac function) and there is no relationship between frequency bands. As a consequence, the power spectrum of white noise is flat. Brownian noise (or random walk noise), on the other hand, presents correlations over (short) time—in a “random walk” pattern, the position of a particle at time *t + 1* will depend of its position at time *t*. Correlations in the time domain, then, have their counterparts in the frequency domain: the power spectra of white noise and Brownian noise are proportional to *f*^*-α*^, with α = 0 and 2, respectively. What is called “pink noise” falls between white noise and Brownian noise with *α* = 1. It is noteworthy that it has been suggested that the power spectrum of spontaneous neural signals follow the general rule *f*^*-α*^, with α close to 1^41^. The exponent *α* was obtained from a linear regression between the PS and frequency (f), as follows: *log(PS) =* α * log(f) + β. The exponent *α* was calculated for each artifact-free epoch of silence, for values in the range between f = 1 and f = 43 Hz. The mean average on all individual β_1_ epochs (i.e. steepness of the slope) was calculated for the tinnitus group and the healthy controls. We compared the steepness of the slope for the two groups for all regions of interest (i.e. 19 sensors and 84 Brodmann areas (see Fig. 1)) using a regression (http://www.ats.ucla.edu/stat/spss/faq/compreg2.htm) and looking at the interaction between the steepness of the slope and group (i.e. t-test).

### Lagged phase coherence

Lagged phase coherence between two sources can be interpreted as the amount of cross-talk between the regions contributing to the source activity^42^. Since the two sources oscillate coherently with a phase lag, the cross-talk can be interpreted as information sharing by axonal transmission. More precisely, the discrete Fourier transform decomposes the signal in a finite series of cosine and sine waves (in-phase and out-of-phase carrier waves, forming the real and imaginary part of the Fourier decomposition) at the Fourier frequencies. The lag of the cosine waves with respect to their sine counterparts is inversely proportional to their frequency and amounts to a quarter of the period; for example, the period of a sinusoidal wave at 10 Hz is 100 ms. The sine is shifted a quarter of a cycle (25 ms) with the respect to the cosine. Then, the lagged phase coherence at 10 Hz indicates coherent oscillations with a 25 ms delay, while at 20 Hz the delay is 12.5 ms, etc. The threshold of significance for a given lagged phase coherence value according to asymptotic results can be found as described by Pascual-Marqui *et al.*^43^, where the definition of lagged phase coherence can be found, as well. This analysis was corrected for the amount of pair wise comparisons using a Bonferroni correction. Time-series of current density were extracted for all regions of interest using sLORETA for all the frequency bands delta (2–3.5 Hz), theta (4–7.5 Hz), alpha1 (8–10 Hz), alpha2 (10–12 Hz), beta1 (13–18 Hz), beta2 (18.5–21 Hz), beta3 (21.5–30 Hz) and gamma (30.5–44 Hz). Power in all 6,239 voxels was normalized to a power of 1 and log transformed at each time point. Region of interest values reflect the log transformed fraction of total power across all voxels separately for specific frequencies.

The lagged phase coherence was determined between pairwise sensors and Brodmann areas independently. This value of the lagged phase coherence signifies the functional connectivity strength between the pairs of sensors or Brodmann areas. Further analyses were performed on the 19 × 19 and 84 × 84 weighted functional connectivity matrices in each of the frequency bands in the two groups. The 19 × 19 matrices consist of 171 undirected edges and the 84 × 84 matrices consist of 3486 undirected edges.

The values of the connectivity strength between each pair-wise combination of sensors or Brodmann areas was then correlated (Pearson’s correlation) with the physical distance between them. The physical distance between nodes (sensors/ Brodmann areas) is calculated by taking the square root of the sum of the square of the difference of the x, y and z coordinates of each of the nodes in MNI space. A linear regression was drawn between the two variables. The significant differences between the steepness of the slopes of regression between the two groups were calculated using the IBM SPSS 22 software.

From the each of the functional connectivity matrices (based on sensors/ Brodmann areas), the following measures were calculated for both the tinnitus and control group at each of the eight frequency bands using the Brain Connectivity Tool Box (BCT) for Matlab^TM^ developed by Rubinov and Sporns in 2010 and updated in 2014^44^.

### Node strength

The strength of a node or node strength is defined as the sum of the weights of all the connections between itself and the remaining nodes in the network. The average node strength of the network is the overall arithmetic average of the strength of all the individual nodes in the network^44^.

### Functional distance and characteristic path length

The functional distance is the length of the shortest path between a pair of nodes. As a first step, the functional connectivity matrix was converted to a connection-length matrix. The functional distance matrix was then computed from the connection-length matrix using Dijkstra’s algorithm^45^. The average shortest path length of the network, termed as the characteristic path length, is the mean of the functional distance matrix where the distance between two nodes is not equal to infinity. The characteristic path length is the measure of global connectivity. Significant differences between the functional distance matrices in the two groups were calculated using Fischer Transformation analysis (Fischer, 1915; Fischer, 1921). A Pearson’s correlation was drawn between the functional distance and the physical distance between all pair-wise nodes (sensors/ Brodmann areas) in the two groups. A linear regression was computed between the two variables and the significant differences in the steepness of the slopes between the two groups were compared using SPSS.

### Clustering coefficient

The clustering coefficient is a node specific measure, which identifies the neighbors of each node and determines the degree of local connectivity of the node with its neighbors. This was calculated by estimating the number of triangles around a node. Clustering coefficients serves as a measure of local connectivity^44^.

### Local efficiency

The local efficiency is also a nodal parameter which characterizes the efficiency of information transfer amongst the neighbors of a particular node. The local efficiency is directly proportional to the clustering coefficient of a node and was calculated from the functional distance matrix. The local efficiency is calculated using the formula given in^44^.

### Cross-Frequency correlations

The shortest path length between pair-wise combination of sensors or Brodmann areas in the lower frequencies (delta, theta, alpha1, alpha2) were correlated with the same measures in the higher frequencies (beta1, beta2, beta3, gamma). The significance of the correlation was tested using Fischer’s Z transformation.

### Statistical Analysis

A repeated-measures ANOVA was performed to analyze the main effect of groups (tinnitus and control) and group x frequency band interaction effect for each of the network connectivity measures mentioned above. Further, simple contrasts were performed to compare the mean network connectivity measures between the two groups in the individual frequency bands. The simple contrasts were analyzed at a significance level of 0.05 (one-tailed). All tests were subject to correction for multiple comparison.

## Results

### 1/f dynamics

**Figure 2:**
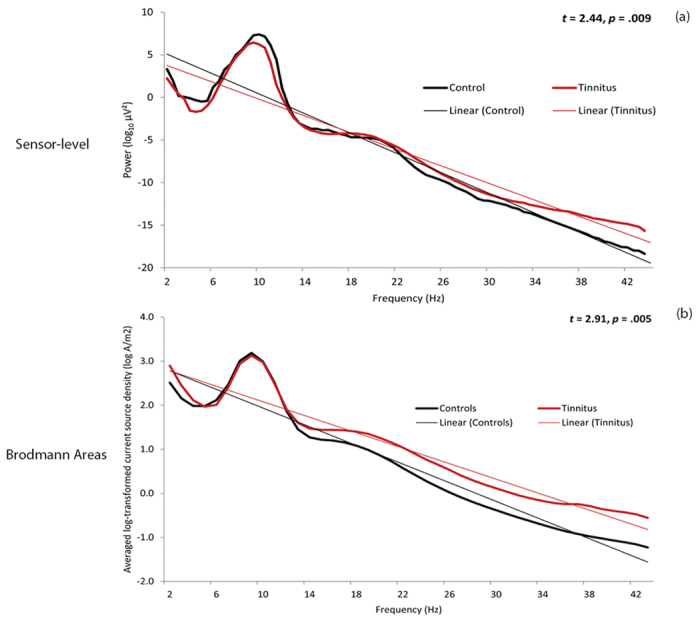
Log-dynamics for the tinnitus group (red) and healthy controls (black) in the (a) Sensor-level and (b) 84 Brodmann area-based analyses.

Comparison between the healthy controls and the tinnitus patients revealed a significant difference in the steepness of the slope at both the sensor level (*t* = 2.44, *p* = 0.009) and source-level (*t =* 2.91, *p =* 0.005). Our data show that the slope of the healthy controls is steeper than the slope of tinnitus patients, indicating a shift to more-randomness, i.e. a whitening of the pink noise structure (see Fig. 2).

### A small-world network

#### Network connectivity analysis on sensor-level data

##### Connectivity strength-network, node and node pairs

**Figure 3:**
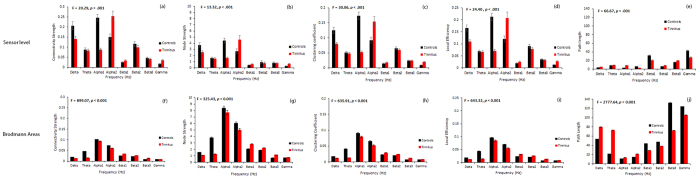
Network connectivity parameters for both (a–e) sensor-level and (f–j) Brodmann Areas-based connectivity analyses in the tinnitus (red) and control (black) groups in the eight frequency bands. The graphs show the mean values of (**a**) Connectivity strength, (**b**) Node strength, (**c**) Clustering coefficient, (**d**) Local efficiency and (**e**) Path length in the sensor-level analysis. (**f**–**j**) correspond to the same measures in the Brodmann Areas-based analysis. The error bars represent standard error.

We observe a significant main effect of groups (*F* = 6.72, *p =* 0.010) showing that the mean connectivity strength of the tinnitus group is significantly different from the control group. This was moderated by the different frequency bands (*F* = 20.29, *p* < 0.001). Performing a simple contrast we observe that the mean connectivity strength of the tinnitus network is significantly lower than that of the control at the delta (*F* = 7.43, *p =* 0.007) and alpha1 (*F* = 73.50, *p* < 0.001) frequency bands. In the alpha2 (*F* = 12.75, *p* < 0.001), beta1 (*F* = 4.46, *p* = 0.035) and gamma (*F* = 17.67, *p* < 0.001) frequency bands tinnitus patients have a significantly higher connectivity strength than that of the controls (Fig. 3). No significant difference in the average connectivity strength was found between the tinnitus and the control groups in the theta (*F* = 0.27, *p* = 0.605), beta2 (*F* = 1.35, *p* = 0.247) and beta3 (*F* = 0.50, *p* = 0.481) bands (Fig. 3).

At the nodal level the mean strength of a node in the tinnitus network is significantly different from the same in the control group (*F* = 4.33, *p =* 0.045). Again, this was moderated by the frequency (*F* = 13.32, *p* < 0.001). Simple contrasts reveal a pattern similar to the network connectivity strength. The mean strength of a node is significantly greater in the control than in the tinnitus network in the delta (*F* = 7.24, *p* = 0.011) and alpha1 (*F* = 51.85, *p* < 0.001) frequency bands and significantly weaker than the tinnitus group at alpha2 (*F =* 5.89, *p* = 0.020), beta1 (*F* = 4.89, *p* = 0.033), and gamma (*F* = 9.47, *p =* 0.004) frequency bands. No significant effect could be obtained for the theta (*F* = 0.27, *p* < 0.604), beta2 (*F* = 1.34, *p* = 0.255) and beta3 (*F* = 0.40, *p* = 0.533) frequency bands (Fig. 3).

##### Clustering Coefficient

The mean clustering coefficient of a node in the tinnitus network is significantly different from the same in the control group (*F* = 13.79, *p =* 0.001). This effect was moderated by the different frequency bands (*F* = 30.60, *p* < 0.001). The mean clustering coefficient is significantly greater in the control than in the tinnitus network in the delta (*F* = 16.51, *p* < 0.001) and alpha1 (*F* = 88.86, *p* < 0.001) frequency bands and significantly weaker than the tinnitus group at alpha2 (*F =* 10.45, *p* = 0.003) and gamma (*F* = 17.62, *p* < 0.001) frequency bands. No significant effect could be obtained for the theta (*F* = 0.42, *p* = 0.523), beta1 (*F* = 2.50, *p* = 0.122), beta2 (*F* = 0.83, *p* = 0.367) and beta3 (*F* = 0.00, *p* = 0.994) frequency bands (Fig. 3).

##### Local Efficiency

The mean efficiency of a node in the tinnitus network is significantly different from the same in the control group (*F* = 8.41, *p =* 0.006). This effect was moderated by the different frequency bands (*F* = 24.40, *p* < 0.001). The difference between the mean local efficiency of the network between the tinnitus and control group closely follows the pattern with the clustering coefficient. The mean local efficiency is significantly greater in the control than in the tinnitus network in the delta (*F* = 13.95, *p* = 0.001) and alpha1 (*F* = 70.27, *p* < 0.001) frequency bands and significantly weaker than the tinnitus group at alpha2 (*F = *10.45, *p* = 0.003), beta1 (*F* = 5.30, *p* = 0.027) and gamma (*F* = 18.22, *p* < 0.001) frequency bands. No significant effect could be obtained for the theta (*F* = 0.39, *p* = 0.536), beta2 (*F* = 1.78, *p* = 0.190) and beta3 (*F* = 0.35, *p* = 0.558) frequency bands (Fig. 3).

##### Functional distance and characteristic path length

**Figure 4:**
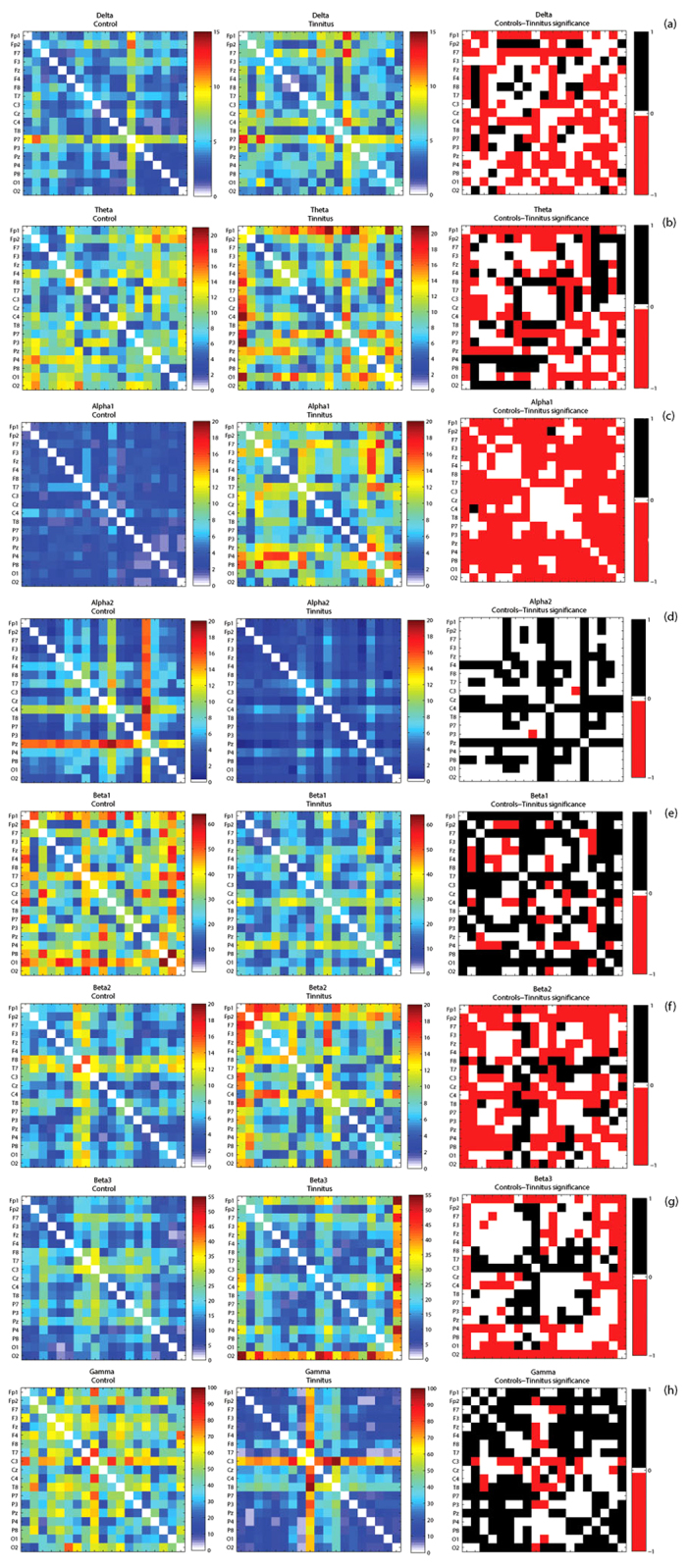
(**a–h**) Correlation matrices of functional distances between pairwise combination of recording electrodes in *left*: Controls and *middle*: Tinnitus, and the *right*: significant differences between these measures in tinnitus and controls calculated by Fischer transformation analysis in (**a**) delta, (**b**) theta, (**c**) alpha1, (**d**) alpha2, (**e**) beta1, (**f**) beta2, (**g**) beta3 and (**h**) gamma frequency bands. In the *right* panel, the areas in red correspond to those pairs of areas between which the functional distance in the tinnitus is significantly longer than the controls, the areas in black correspond to those pairs of areas between which the functional distance in controls is significantly longer than tinnitus and the areas in white are those pairs of areas between which there is no significant difference in functional distance between tinnitus and controls.

**Figure 5:**
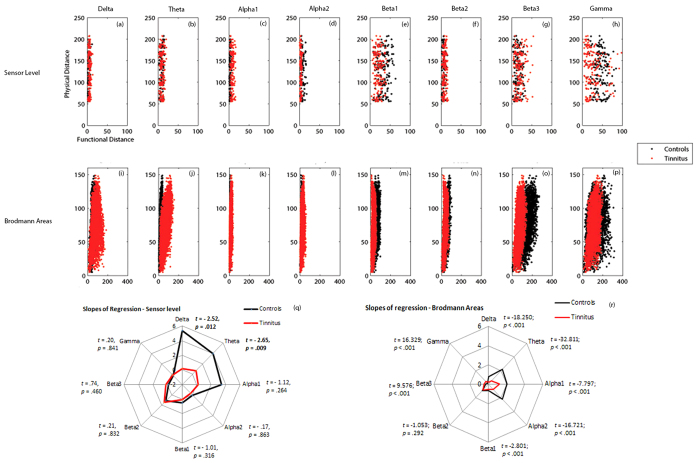
Correlation of physical distances and functional distances between pair-wise (a–h) sensors and (i–p) Brodmann areas in controls (black) and tinnitus (red) in delta, theta, alpha1, alpha2, beta1, beta2, beta3 and gamma frequency bands. Comparison of the slopes of regression of the physical distance over functional distance between pairwise combination of (**q**) sensors and (**r**) Brodmann areas in controls (black) and tinnitus (red) subjects in the eight frequency bands.

The mean functional distance in the tinnitus network is significantly different from the same in the control group (*F* = 52.09, *p* < 0.001). This effect was moderated by the different frequency bands (*F* = 66.67, *p* < 0.001). The mean functional distance or the characteristic path length of the network is significantly lesser in the control than in the tinnitus network in the delta (*F* = 33.22, *p* < 0.001), theta (*F* = 5.43, *p* = 0.020), alpha1 (*F* = 352.21, *p* < 0.001), beta2 (*F* = 26.85, *p* < 0.001) and beta3 (*F* = 14.67, *p* < 0.001) frequency bands and significantly greater than the tinnitus group in the alpha2 (*F = *97.77, *p* < 0.001), beta1 (*F* = 86.00, *p* < 0.001) and gamma (*F* = 67.71, *p* < 0.001) frequency bands. We also observe that the functional distance between pair-wise combination of sensors is significantly longer in the tinnitus in the delta, theta, alpha1, beta2 and beta3 frequency bands. The functional distance between pair-wise combination of sensors is significantly longer in the controls in the alpha2, beta1 and gamma frequency bands. In the alpha2 frequency band, there are also some areas between which there is no significant difference in the functional distance across the two groups (Fig. 4). There exists a direct relationship between the shortest functional distance and the anatomical distance between pairs of sensors in the different frequency bands also moderated by the frequency bands. The functional distance between anatomically closer areas in the tinnitus network are significantly longer than the controls in only the delta and theta frequency bands and no significant difference exists in the other frequency bands (see Fig. 5).

#### Connectivity analysis on a source-level data

##### Connectivity strength-network, node and node pairs

We observe a significant main effect of groups (*F* = 83.13, *p* < 0.001) indicating that the mean connectivity strength of the tinnitus group is significantly different from the control group. This effect was moderated by the different frequency bands (*F* = 276.26, *p* < 0.001). A simple contrast shows that the mean connectivity strength of the tinnitus network is significantly lower than that of the control at delta (*F* = 500.27, *p* < 0.001), theta (*F* = 2947.17, *p* < 0.001), alpha1 (*F* = 19.39, *p* < 0.001) and alpha2 (*F* = 99.12, *p* < 0.001) frequency bands. For the beta1 (*F* = 241.19, *p* < 0.001), beta2 (*F* = 113.66, *p* < 0.001), beta3 (*F* = 533.22, *p* < 0.001) and gamma (*F* = 6.98, *p* = 0.008) frequency bands tinnitus patients have a significantly higher connectivity strength than that of the controls (Fig. 3).

At the nodal level the mean strength of a node in the tinnitus network is significantly different from the same in the control group (*F* = 15.10, *p* < 0.001). Again, this was moderated by the frequency (*F* = 325.43, *p* < 0.001). Simple contrasts reveal a pattern similar to the network connectivity strength. The mean strength of a node in the tinnitus network is significantly weaker than the controls in delta (*F* = 124.62, *p* < 0.001), theta (*F* = 863.65, *p* < 0.001) and alpha2 (*F = *12.83, *p* < 0.001) frequency band and significantly stronger than the controls at beta1 (*F* = 47.80, *p* < 0.001), beta2 (*F* = 36.36, *p* < 0.001) and beta3 (*F* = 110.97, *p* < 0.001). No significant effect could be obtained for the alpha1 (*F* = 2.51, *p =* 0.115) and gamma (*F* = 1.15, *p = *0.285) frequency bands (Fig. 3).

##### Clustering Coefficient

We observe that the mean clustering coefficient of the tinnitus network is significantly different from the control network (*F* = 54.12, *p* < 0.001) and that this effect is moderated by the different frequency bands (*F* = 84.81, *p* < 0.001). Simple contrasts at individual frequency bands reveal that the clustering coefficient of the tinnitus network is significantly lower than that of the control at delta (*F* = 322.17, *p* < 0.001), theta (*F* = 2062.80, *p* < 0.001), alpha1 (*F* = 13.36, *p* < 0.001) and alpha2 (*F* = 42.04, *p* < 0.001) frequency bands and is significantly greater than the control network at beta1 (*F* = 80.18, *p* < 0.001), beta2 (*F* = 83.23, *p* < 0.001), beta3 (*F* = 324.96, *p* < 0.001) and gamma (*F = *8.18, *p *= 0.005) frequency bands (Fig. 3).

##### Local Efficiency

The mean local efficiency of a node closely follows the clustering coefficient of the network because of its direct relationship. The mean local efficiency of a node in the tinnitus network is significantly different from that of the controls (*F* = 25.28, *p* < 0.001) and is moderated by the frequency bands (*F* = 75.73, *p* < 0.001). A simple contrast reveals that the mean local efficiency of a node in the tinnitus network is significantly lower than that of the controls at delta (*F* = 292.43, *p* < 0.001), theta (*F* = 2070.11, *p* < 0.001), alpha1 (*F* = 10.81, *p* = 0.001) and alpha2 (*F* = 4.03, *p* = 0.046) frequency bands and is significantly higher than that of the controls at beta1 (*F* = 115.08, *p* < 0.001), beta2 (*F* = 90.23, *p* < 0.001), beta3 (*F* = 336.25, *p* < 0.001) and gamma (*F* = 6.08, *p* = 0.015) frequency bands (Fig. 3).

##### Functional distance and characteristic path length

**Figure 6:**
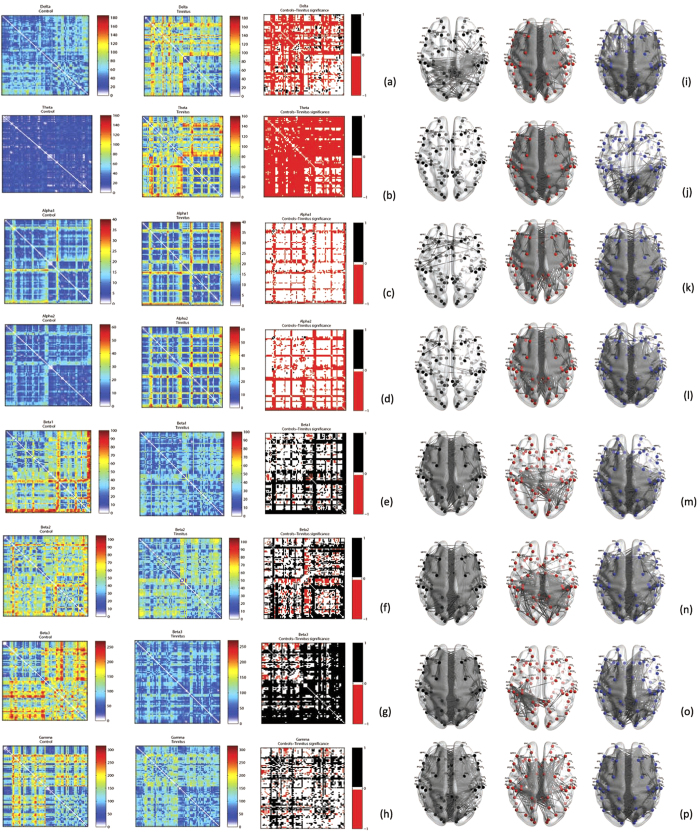
(**a–h**) Correlation matrices of functional distances between pairwise combination of Brodmann areas in *left*: Controls and *middle*: Tinnitus, and the *right*: significant differences between these measures in tinnitus and controls calculated by Fischer transformation analysis in (**a**) delta, (**b**) theta, (**c**) alpha1, (**d**) alpha2, (**e**) beta1, (**f**) beta2, (**g**) beta3 and (**h**) gamma frequency bands. In the *right* panel, the areas in red correspond to those pairs of areas between which the functional distance in the tinnitus is significantly longer than the controls, the areas in black correspond to those pairs of areas between which the functional distance in controls is significantly longer than tinnitus and the areas in white are those pairs of areas between which there is no significant difference in functional distance between tinnitus and controls. (**i–p**). The network containing the actual connections whose functional distance between Brodmann areas are *left*: significantly longer in controls, *middle*: significantly longer in tinnitus subjects and *right*: not significantly different between the two groups in (**i**) delta, (**j**) theta, (**k**) alpha1, (**l**) alpha2, (**m**) beta1, (**n**) beta2, (**o**) beta3 and (**p**) gamma frequency bands.

A significant main effect of groups (*F* = 42.97, *p* < 0.001) moderated by frequency (*F* = 3546.18, *p* < 0.001) was observed for the characteristic path length indicating that the characteristic path length of the tinnitus network is significantly different from the controls and this difference varies by frequency bands. Simple contrasts reveal that the characteristic path length of the tinnitus network is significantly longer than that of the controls in the lower frequency bands, such as delta (*F* = 1991.67, *p* < 0.001), theta (*F* = 9868.59, *p* < 0.001), alpha1 (*F* = 219.75, *p* < 0.001) and alpha2 (*F* = 960.08, *p* < 0.001) and is significantly shorter than that of the controls in the higher frequency bands, such as beta1 (*F* = 1806.39, *p* < 0.001), beta2 (*F* = 644.50, *p* < 0.001), beta3 (*F* = 4168.49, *p* < 0.001) and gamma (*F* = 294.21, *p* < 0.001) bands (Fig. 3). We also observe that the shortest distance between selective pairs of Brodmann areas in the tinnitus network are significantly longer in the lower frequency bands (delta, theta, alpha1, alpha2) and significantly shorter in the higher frequency bands (beta1, beta2, beta3, gamma) (Fig. 6). There exists a direct relationship between the shortest functional distance and the anatomical distance between pairs of Brodmann areas in the different frequency bands, which is also moderated by the oscillatory frequencies. The functional distance between anatomically closer areas in the tinnitus network are significantly longer in most of the frequency bands (delta, theta, alpha1, alpha2, beta1) and significantly shorter in beta3 and gamma compared to the controls (see Fig. 5).

##### Cross-Frequency correlations

No significant change in the cross-frequency Pearson correlation of the shortest path length was observed between the tinnitus and control groups in the sensor-level connectivity analysis (Table 2). However, we observe a significant change in the Pearson correlation between the shortest path length amongst pairs of Brodmann areas in the low frequency bands with the same in high frequency bands in the tinnitus group. The individual correlations of each of the low frequency bands (delta, theta, alpha1 and alpha2) with each of the high frequency bands (Beta1, Beta2 and Beta3) are significantly lower in the tinnitus group. However, the correlations of delta, theta and alpha1 with gamma are significantly greater in the tinnitus group. No significant change in correlation between alpha2 and gamma bands was observed (Table 3).

## Discussion

The brain has been considered a Bayesian prediction machine that updates its memory-based predictions through active sensory exploration of the environment^46^,^47^. This concept has been translated to tinnitus, as well^48^. The oscillatory activity related to auditory predictions has been recently identified^49^: delta-beta coupled oscillations underpin prediction accuracy^49^, and (Bayesian) updating of the predictions is processed by the alpha-band (10–14 Hz)^49^. Predicting ‘when’ an auditory stimulus arrives predominantly involves low-frequency delta and theta oscillations, predicting ‘what’ is processed by gamma and beta oscillations^50^. Beta oscillations likely underlie a top-down flow of information, whereas gamma oscillations could be generated bottom-up^50^. As predictions are transmitted in a top-down ‘backward’ manner, using mainly the beta band, prediction errors could be propagated in the gamma band in a bottom-up feed-forward manner^50^. Updating of the predictions via attention-based scanning of the environment^51^, on the other hand, is linked to alpha oscillations^49^,^51^. Thus, by transferring these findings to tinnitus, one could speculate that increased gamma activity in tinnitus would be related to a deafferentation related (thalamocortical column specific spatial mismatch) prediction error, and the nesting on theta or delta related to its temporal prediction. In other words, gamma activity could reflect any change in the auditory environment, as this induces a prediction error.

Complex adaptive systems are by definition complex, i.e. made up of many components, connected in a specific way, typically composed of networks that are able to process and withstand a broad range of stresses and typically generate complex output signals that have a 1/f decay of the power spectra^2^,^3^. Their main characteristics is adaptiveness, i.e. the capacity to change and learn from experience, giving them resilience in the face of perturbation (homeostasis)^2^,^3^. They typically have a small world topology and are noisy, i.e. have some randomness embedded in the system^1^, which permits the system to learn, i.e. adapt.

The brain has such a small world topology, which is an intermediate topology between a lattice (=regular) structure and a completely random structure^24^. Small world topologies have a small average shortest path length, and a large clustering coefficient^21^,^52^. The large clustering coefficient results in the formation of subnetworks, typical of small world networks. The small path length is due to the presence of many hubs, i.e. densely connected nodes. Lattice or large world networks are completely determined, in that they always generate the same response to a stimulus, making this structure very efficient, but also very predictable and thus not very good for survival^53^. The other extreme, a random topology, is inefficient in that it generates a different response to identical stimuli, due to its inherent memory-less randomness. Random graphs exhibit a small average shortest path length along with a small clustering coefficient^21^. The brain, with its small world topology has the capacity to adjust to a changing environment by adding long range connections to the predominant short range connections present in lattice structures^14^,^24^. Losing long range connections results in the transition of small worldness to a more regular or lattice topology. Adding long range connections to a small world topology, so that all nodes have a similar amount of connections results in a transition from small worldness to a more random topology. The brain has been described as a hierarchical scale-free network^13^,^16^,^18^,^19^,^54^,^55^,^56^ and scale-free networks are small world networks^57^, more specifically, ultra-small world networks^58^.

Deviations from the 1/f pattern and related network changes have been associated with disease. The aim of current research was to identify whether tinnitus is an emergent property of an altered network topology, which could be recognized by a specific oscillatory pattern and connectivity signature differing from healthy subjects. Our results are noteworthy for various reasons. First, they demonstrate a clear shift from a typical 1/f pink noise pattern in healthy controls toward a more white noise pattern in tinnitus patients. This shift can be observed at both the sensor and source level. Second, we identified differences in the connectivity networks of the brain in individuals with and without tinnitus. Although the differences in network connectivity measures between the two groups in the different frequency bands do not follow the exact same trend at senor and source level, the current study reports an overall change in the network parameters in the tinnitus group compared to the control group for connectivity strength, node strength, clustering coefficient and local efficiency. The differences in the trend can be attributed to the fact that EEG is a scalp potential and the signal collected from one sensor may be the sum of the signal from different sources. Also, the source-localization is a statistical estimation of the sources and hence is subject to technical limitations in accurate estimation.

At both the sensor and source level, we see a shift from pink towards a more white noise (=more random signal) in tinnitus patients. This suggests more randomness in the network and relatively more high frequency oscillatory activity. A similar shift is seen in schizophrenia^9^, anxiety^10^ and Alzheimer^8^. The 1/f pink noise behavior of spontaneous oscillations has been interpreted within the theory of self-organized criticality^41^. Pink noise seems to be the optimal transition between order and randomness^59^, a state of supple regulation which permits a system to efficiently respond to stimuli, but then return to baseline^60^. In contrast, in tinnitus patients a shift-to-randomness may imply less coordinated signal organization at the local level of possibly neural circuits.

Previous research already suggested that brain dynamics are strongly influenced by the mean number of long range connections^61^,^62^, characteristic of small world networks. Both our sensor and source-level results show an increase in the characteristic path length, decrease in long distance connectivity and an increase in connectivity strength between anatomically closer areas, suggesting a shift of the tinnitus network towards a more lattice (i.e., regular) topology in the lower oscillatory frequencies^21^,^63^. This creates a high local efficiency at a low cost, i.e. is energy efficient. However, the system loses flexibility or adaptiveness, which is clinically expressed as the persistence of the tinnitus sound. It is known that when cognitive demand is lower, brain networks ‘relax’ into a more clustered and less costly lattice-like configuration^64^. So, it is possible that the tinnitus brain is getting more efficiently organized to focus only on the tinnitus sound, possibly due to a paradoxical salience attached to the sound^65^. Our data support this idea, as we see for the theta band specific connections between the right temporal cortex and bilateral parietal areas and also the fronto-limbic connections that are still intact in the tinnitus patients. Indeed in chronic tinnitus, functional connectivity is increased between the parahippocampal area (i.e., auditory memory), the auditory cortex and the cingulate and insula even though general connectivity decreases^36^, and this insula and cingulate activity is associated with the loudness perception^66^. Increased connectivity in the network of connections between the parahippocampus and the auditory cortex was reported in resting state functional connectivity as well^67^. Further studies also showed an increased connectivity with subcortical areas such as the brainstem, cerebellum, right basal ganglia and nucleus accumbens for the tinnitus group in contrast to the control group^68^. The relationship with sub-cortical structures cannot be explored in the current study due to the limitations of the modeling technique for estimating sources of the EEG generators. For the higher frequencies, we see a decrease in absolute path length, increase in long distance connectivity and decrease in shortest distance between pairs of brain areas that are anatomically further away in both sensor level and source level analysis. This could allude to the shift in the tinnitus network to a more random topology for the higher frequencies. It is known that network efficiency for integrative processing is being maximized by a random topology^24^. This suggests that the brain randomly connects to other parts of the brain, hypothetically in an attempt to retrieve the deafferentation related missing information from wherever it can. Indeed, it has been suggested that synchronization of distributed focal gamma band activity might bind different aspects of brain processing into one coherent unified percept, both in the visual^69^ and auditory systems as it pertains to tinnitus^4^.

The higher frequencies usually convey information at short distances and lower frequencies at long distances^70^,^71^. It is further known that frequency coupling between high and low frequencies provides a mechanism for the control of localized neural processing by distributed brain networks^71^,^72^. Our source-level analysis further suggests that the long distance connectivity in the gamma frequency band seems to be in parallel with the long distance connectivity in the delta, theta, alpha1 and apha2 frequency bands for the tinnitus group. This is evidenced by a significantly stronger correlation of functional distance between delta and gamma, theta and gamma and alpha1 and gamma in tinnitus patients in comparison to healthy controls. This might indicate that there could be a common path in the transfer of information with the lower frequencies that is exclusive to the tinnitus network. This pattern is however not seen at the sensor-level. Cross-frequency coupling within the auditory cortex has been documented in tinnitus^4^,^73^, as well as between tinnitus related areas^66^,^74^. In addition, the role of low-frequency modulation of gamma power in sustained auditory attention (i.e., sustained focus on the tinnitus) has been shown, as demonstrated by the strength of gamma–theta coupling across frontal and posterior areas^75^,^76^. Sustained attention in the tinnitus population was also reported by resting state functional MRI studies^77^,^78^. An increased connectivity between the auditory cortex and the dorsal attention network (intraprietal sulci, ventral precentral gyrus, middle frontal gyrus, and frontal eye fields)^77^ also confirm the idea of sustained attention to the tinnitus percept in order to suppress the phantom sound^78^.

The intriguing results of this study demonstrate that low frequency communication via delta and theta carrier waves shows a transition from a small world topology to a more regular i.e. less flexible topology, whereas the information carrying high frequencies (beta and gamma) transitions from a small world topology to a more random topology. This apparent topological dissociation between low and high oscillatory connectivity could hypothetically represent different adaptive strategies to the auditory deprivation. On the one hand theta carrier waves lose their long range connectivity, resulting in a less flexible brain topology. One can hypothesize the brain limits its filling-in mechanisms to a quasi-stable state in which the missing information is pulled from auditory memory^79^,^80^. On the other hand, gamma activity has been linked to a bottom-up prediction error resulting from omitted information^4^,^66^ and has been linked to representing the tinnitus sound, and even its loudness perception^66^. The gamma random connectivity could represent a distribution of a bottom-up auditory prediction error in the brain. These random connections increase the likelihood that the tinnitus-related gamma band activity connects to the consciousness supporting networks^81^, also known as the global workspace^82^,^83^, an essential requirement for the gamma band activity to be pushed to awareness. But, one can also hypothesize that by randomly broadcasting the prediction error to wider brain areas, other solutions could be found for the prediction error, e.g. by multisensory congruence, looking for example for visual or other sensory stimuli that could help solve the prediction error. This could explain why in many tinnitus studies the functional connectivity with the visual cortex is altered^84^,^85^,^86^.

In spite of the superior temporal resolution of EEG and statistical power due to the large sample sizes of the two groups, a major drawback of the study is that the control population is not matched for hearing loss. Another drawback of the study is that the functional connectivity is calculated between Brodmann areas estimated from resting state EEG recorded using 19 electrodes. Thus the spatial resolution is not as precise as observed in resting state or block-design fMRI studies. This combined with the limitations of the modeling technique also restricts us to only examining cortical structures and not subcortical structures which are also reported to play an important role in tinnitus. However, the current study paves way for further research which uses graph theory with source-localized resting state EEG for studying tinnitus wherein much stricter conditions could be placed on the selection of the patient and control population.

## Conclusion

The brain seems to reorganize its topology in two ways in tinnitus patients. The low frequency carrier wave oscillations reorganize in to a more regular or lattice structure, whereas the higher information carrying oscillations seem to restructure more into a random topology. We hypothesize these topological changes represent two opposite attempts to reduce the uncertainty linked to the auditory deafferentation, usually associated with tinnitus.

## Additional Information

**How to cite this article**: Mohan, A. *et al.* Graph theoretical analysis of brain connectivity in phantom sound perception. *Sci. Rep.* **6**, 19683; doi: 10.1038/srep19683 (2016).

## Figures and Tables

**Table 1:** Tinnitus characteristics.

**Ear**	
* Unilateral*	114
* Bilateral*	197
**Tone**	
* Pure tone*	118
* Noise Like*	193
**TQ**	
* Mean*	39.37
* Sd*	17.59
**Tinnitus Frequency (Hz)**	
* Mean*	5143
* Sd*	3183
**Hearing loss at the Tinnitus Frequency (dB SL)**	
* Mean*	7.85
* Sd*	8.78

**Table 2:** Pearson correlations of functional distance of pair-wise sensors at lower frequencies (delta, theta, alpha1 and alpha2) with the same pair-wise brain areas at higher frequencies (beta1, beta2, beta3 and gamma).

Functional Distance												
	Delta			Theta			Alpha1			Alpha2		
	C	T	∆	C	T	∆	C	T	∆	C	T	∆
Beta1	−0.09	0.21	−1.19	−0.07	0.19	−1.05	0.07	0.11	−0.16	−0.02	0.32	−1.38
Beta2	0.12	0.04	0.30	−0.11	0.08	−0.75	0.11	−0.05	0.65	−0.10	0.03	−0.52
Beta3	0.19	0.17	0.07	−0.29	0.09	−1.56	0.03	0.10	−0.29	0.17	−0.02	0.75
Gamma	−0.04	−0.08	0.18	−0.08	−0.24	0.67	0.03	−0.18	0.84	−0.09	0.22	−1.23

**Table 3:** Pearson correlations of functional distance of pair-wise brain areas at lower frequencies (delta, theta, alpha1 and alpha2) with the same pair-wise brain areas at higher frequencies (beta1, beta2, beta3 and gamma).

Functional Distance												
	Delta			Theta			Alpha1			Alpha2		
	C	T	∆	C	T	∆	C	T	∆	C	T	∆
Beta1	0.34	−0.07	**7.69**	0.50	0.06	**8.87**	0.64	−0.13	**16.11**	0.47	−0.20	**12.92**
Beta2	0.45	−0.12	**10.97**	0.59	0.16	**9.36**	0.39	−0.09	**9.10**	0.43	−0.10	**10.15**
Beta3	0.46	−0.11	**11.01**	0.40	0.03	**7.13**	0.32	−0.24	**10.45**	0.36	−0.25	**11.46**
Gamma	0.27	0.38	−**2.23**	0.31	0.47	−**3.43**	0.07	0.24	−**3.17**	0.26	0.32	−1.19

C: healthy controls; T: Tinnitus patients; ∆ comparison between C and T.
